# Can I Stay, or Must I Go Now? A Cohort Study of Discharge Appeals in a Post-Acute Skilled Nursing Facility

**DOI:** 10.1016/j.jamda.2025.106108

**Published:** 2026-02-14

**Authors:** W. James Deardorff, Grant Tominaga, James D. Harrison, Himali Weerahandi, Matthew J. Miller, Michi Yukawa, Kenneth Lam

**Affiliations:** a Division of Geriatrics, University of California San Francisco, San Francisco, CA, USA; b Philip R. Lee Institute for Health Policy Studies, University of California San Francisco, San Francisco, CA, USA; c Division of Hospital Medicine, University of California San Francisco, San Francisco, CA, USA; d Department of Physical Therapy and Rehabilitation Science, University of California San Francisco, San Francisco, CA, USA; e Division of Geriatric Medicine, Department of Medicine, University of Colorado Anschutz Medical Campus, Aurora, CO, USA; f Division of Hospital Medicine, Department of Medicine, University of Colorado Anschutz Medical Campus, Aurora, CO, USA

**Keywords:** Skilled nursing facility, post-acute care, discharge appeal, Medicare Advantage

## Abstract

**Objectives:**

Patients admitted to a skilled nursing facility (SNF) for short-term rehabilitation after hospitalization often feel unprepared to return home and may appeal discharge dates set by SNFs and/or insurers. There are additional concerns that Medicare Advantage (MA) insurers may be more aggressive about discharging patients earlier compared with traditional Medicare. Yet, little has been published on the characteristics of patients who appeal and their outcomes.

**Design:**

Retrospective cohort study.

**Setting and Participants:**

Participants included patients admitted to a single SNF after hospitalization from March 1, 2024, to March 31, 2025, who filed discharge appeals.

**Methods:**

We collected information via chart reviews on patient demographics (eg, age, insurance coverage), comorbidities, function scores, and documented reasons for appeal. We also identified outcomes following appeal (eg, 30-day rehospitalization, death).

**Results:**

Of 453 eligible SNF admissions, 47 (10.4%) patients filed 58 appeals [mean age 79.3 (SD = 10.6), 25 (53.2%) female, 9 (19.1%) Asian, 7 (14.9%) Black, 20 (42.6%) in traditional Medicare, 27 (57.4%) in MA]. Median (IQR) time from SNF admission to first appeal was 19.0 (15.0–30.5) days. Eleven patients (23.4%) won their appeals. The median (IQR) time from first appeal to discharge was 8 (7–13) and 4 (3–8) days among patients who won their appeals vs those who lost their final appeal, respectively. The 30-day rehospitalization and 30-day mortality rates among those who won their appeals were 0% (n = 0 of 11) and 18.2% (n = 2 of 11), respectively. Among those who lost their final appeal, rates were 27.8% (n = 10 of 36) and 0% (n = 0 of 36), respectively. The most common reason for appealing was patient and/or family/caregiver concern about discharge readiness (n = 28, 59.6%).

**Conclusions and Implications:**

In this single-SNF study, 10% of post-acute patients appealed their discharge, commonly citing concerns about discharge readiness, with most ultimately losing their final appeal. This study lays the groundwork for future research examining appeals processes and outcomes on a broader scale.

Roughly 20% of hospitalized older adults are discharged to a skilled nursing facility (SNF) for short-term nursing and rehabilitative services.^1^ Although patients go to SNFs to recover independence, many subsequently experience adverse outcomes, such as rehospitalization and prolonged periods of functional impairment.^1-3^ Therefore, the discharge process from an SNF to home is frequently a distressing time for patients and caregivers who may feel unprepared and unsafe to return home.^4,5^

Discharge dates for short-stay SNF admissions may be set by the SNF clinical team, often in conversation with patients and caregivers, or by insurance companies, who may be further removed from the social and medical circumstances of patients. Patients and caregivers who disagree with the discharge date may file an appeal through a Beneficiary and Family Centered Care-Quality Improvement Organization, where an independent reviewer decides whether the SNF services should continue.^6^ If the appeal is successful, covered services continue until a new discharge date is established. If a patient and/or caregiver disagrees with subsequent discharge dates, they may file additional appeals during the SNF stay.

To date, little empirical research has been published on discharge appeals at SNFs, including their frequency and success rate, characteristics of appellants, and outcomes following an appeal. One concern is that prematurely discharging patients from an SNF may lead to adverse outcomes, such as rehospitalization and death.^7^ This issue was brought to the forefront when a class action lawsuit was filed in 2023 against UnitedHealth Group, the largest insurer in the United States, for allegedly shortening the lengths of stay of SNF patients enrolled in Medicare Advantage (MA) plans based on an algorithm rather than clinician judgment.^8,9^ Plaintiffs argued that premature discharges left patients scrambling to arrange care and at risk for adverse outcomes.

Given the lack of published research on this topic, we conducted an exploratory study at a single SNF to document the characteristics of SNF patients who appealed their discharge and report outcomes of patients who won or lost their appeals. The goal of this study was to inform the design of larger-scale studies that more comprehensively examine the discharge appeal process.

## Methods

### Study Design and Participants

We conducted a retrospective cohort study using chart review of post-acute SNF patients who appealed their discharge. We included patients discharged from a quaternary academic hospital and subsequently admitted to a single 100+-bed urban nonprofit SNF in northern California from March 1, 2024, to March 31, 2025. During the study period, the SNF had 4 out of 5 stars on short-stay quality measures on Nursing Home Compare, and ~65% of short-stay patients were enrolled in traditional Medicare. As part of usual operations, case managers are notified by email of appeals. We used patient identifiers in these emails to identify our cohort. The study was reviewed and approved by the University of California, San Francisco, Committee on Human Research.

### Data Source

We reviewed data from the SNF electronic health record (EHR; PointClickCare), including notes from clinical staff (eg, case managers, social workers, physicians, nurse practitioners, nurses, and physical and occupational therapists) and Minimum Data Set (MDS) assessments. The MDS is a comprehensive assessment completed on admission and discharge for short-stay residents.^10^ We ascertained outcomes using data from the academic hospital’s EHR linked by unique patient identifiers.

### Characteristics of Persons Who Appeal

To characterize persons who appealed, we considered demographic and clinical factors that could influence appeal and clinical outcomes after SNF discharge.^11^ Demographics included age, sex, marital status, and insurance type (eg, traditional Medicare and MA). Clinical factors included comorbidities, primary medical diagnosis on the MDS, and hospital length of stay. Functional factors included cognition (based on the MDS Cognitive Function Scale), bladder and bowel incontinence, and functional status.

### Outcomes and Reasons for Appeals

We collected the time from SNF admission to first appeal, the time from first appeal to SNF discharge, the outcome of the appeal (win/loss), total SNF length of stay, and discharge location. Clinical outcomes included functional status over time, 30-day rehospitalization, and mortality at 30 days, 3 months, and 6 months. Functional status, which was only available for 36 of the 47 patients, was measured using the Patient Driven Payment Model (PDPM) function score at admission, time of appeal, and at discharge. The score was determined from routine physical and occupational therapy assessments, ranging from 0 to 24, with higher scores indicating better functioning (Supplementary Methods).^12^ We classified survival based on documented deaths or documented survival inferred from ongoing clinical events in the linked hospital EHR; if a patient had no documented clinical events in the time frame (eg, over 30 days), we considered the outcome as missing.

We also hierarchically categorized the reason for appeal as (1) SNF clinician concern about clinical status, (2) patient and/or family/caregiver concern about discharge readiness, or (3) no reason documented (Supplementary Table 1). This categorization schema was developed by reviewing clinical documentation from case managers, social workers, physicians, and nurse practitioners pertaining to the appeal. Investigators (W.J.D., K.L.) independently categorized documentation with 90% concordance. Discrepancies were resolved via consensus.

### Data Analysis

We report summary-level statistics, including mean [standard deviation (SD)] and median [interquartile range (IQR)] for continuous variables and frequency for categorical variables. We categorized patients into 2 groups based on the outcome of their final appeal: patients who won their appeals and patients who lost their final appeal. The latter group encompasses patients who lost their only appeal or, for patients with multiple appeals, lost their final appeal before SNF discharge. We used this distinction because patients who lost their final appeal likely represent those who were discharged despite having ongoing concerns. We also explored whether time to event outcomes (eg, time from appeal to discharge) differed when (1) analyzing by appeal rather than by patient and (2) analyzing only patients who filed multiple appeals during a single SNF stay. Given the exploratory nature of the study, formal statistical significance testing was not conducted. Statistical analyses were performed using R version 4.4.1 (R Project for Statistical Computing).

## Results

### Cohort Characteristics

**Fig. 1.**
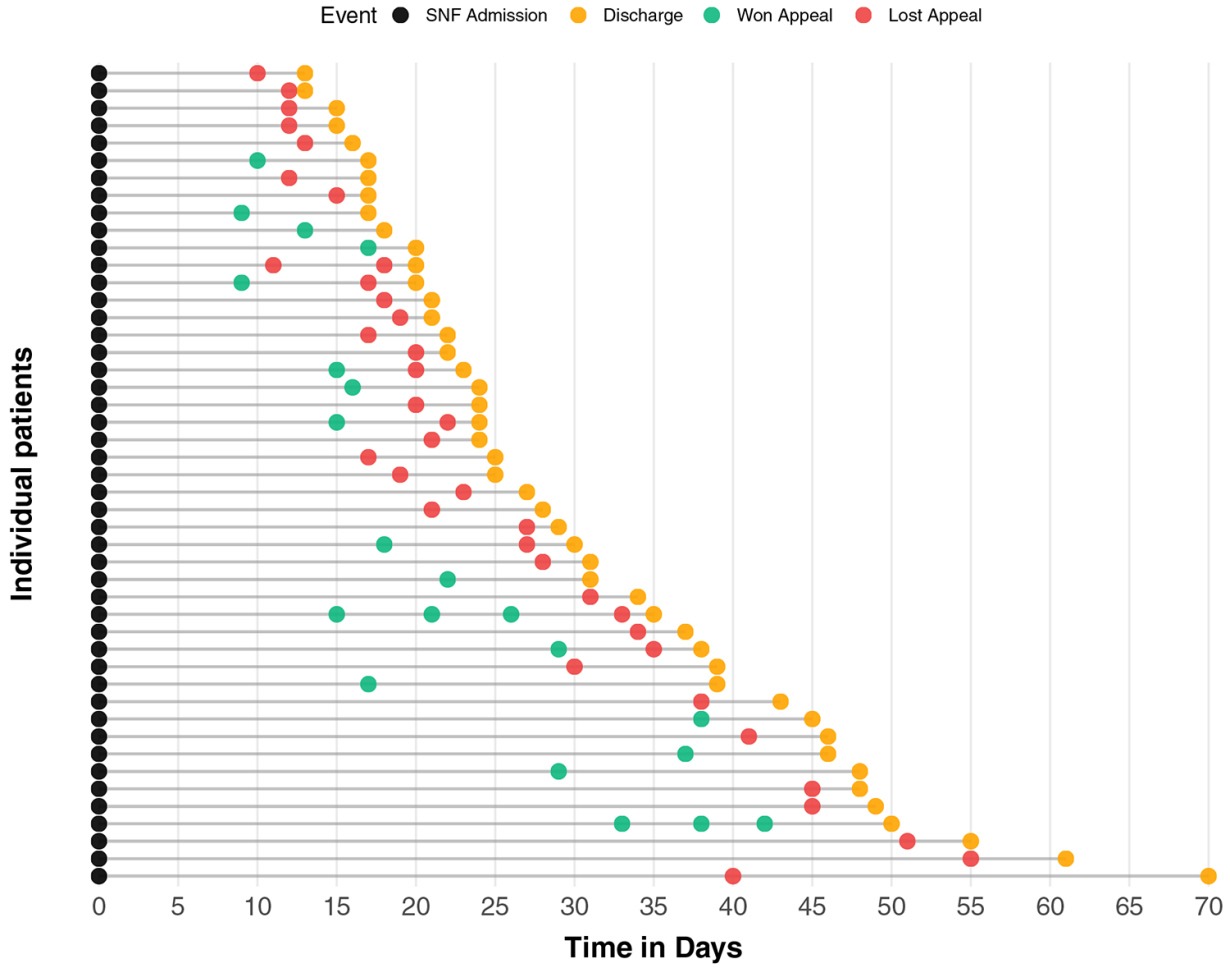
Timeline of events during an SNF admission for the 47 patients who appealed their discharge date in the study. In this figure, each line represents an individual patient. Each circle represents an event that occurred during the SNF admission. The first black circle represents the SNF admission, and the final orange circle represents the SNF discharge date. Circles in-between represent when an appeal was filed and the outcome of the appeal, with green circles indicating that the appeal was won and red circles indicating that the appeal was lost.

During the study period, there were 453 eligible short-term SNF admissions. The final cohort included 47 patients (10.4%) who appealed a combined 58 times, averaging 1.23 (SD = 0.60) per appellant (Figure 1 and Supplementary Figure 1). Eight patients (17.0%) filed multiple appeals during the SNF stay. Eleven patients (23.4%) won all their appeals (10 patients filed 1 appeal and won, and 1 patient filed 3 appeals and won all 3), and 36 patients (76.6%) lost their final appeal (29 patients filed 1 appeal and lost, and 7 patients filed multiple appeals and lost their final appeal).

The mean age of appellants was 79.3 (SD = 10.6), 25 (53.2%) were women, 9 (19.1%) were Asian, 7 (14.9%) were Black, and 28 (59.6%) were White (Table 1). Twenty-seven (57.4%) were enrolled in MA plans, and 20 (42.6%) were enrolled in traditional Medicare. The most common reason for admission was orthopedic surgery (eg, joint replacement, spinal surgery, fracture) (n = 20, 42.6%). The mean number of comorbidities was 2.9 (SD = 1.7).

### Outcomes

The median time to first appeal was 19 days (IQR 15.0–30.5) and was similar between those who won their appeals and those who lost their final appeal (median 17.0 vs 19.5 days) (Table 1). The median time from appeal to discharge was 4 days longer for those who won their appeals compared with those who lost their final appeal (median 8 vs 4 days). Total SNF length of stay (median 25 days, IQR 20–39) was similar between the 2 groups. Results were similar when analyzing by appeal (n = 58; Supplementary Table 2) or when examining only those who filed multiple appeals (n = 8; Supplementary Table 3).

Most patients were discharged home to their previous living environment (n = 30, 63.8%) (Table 1). Overall, the 30-day rehospitalization rate was 21.3% (n = 10), and the 3-month mortality rate was 12.8% (n = 6) (Table 1). The 30-day rehospitalization rate among patients who lost their final appeal was 27.8% (n = 10) compared with no rehospitalizations in those who won their appeals. Two patients who won their appeals died within 30 days (18.2%) compared with no patients who lost their final appeal.

**Fig. 2.**
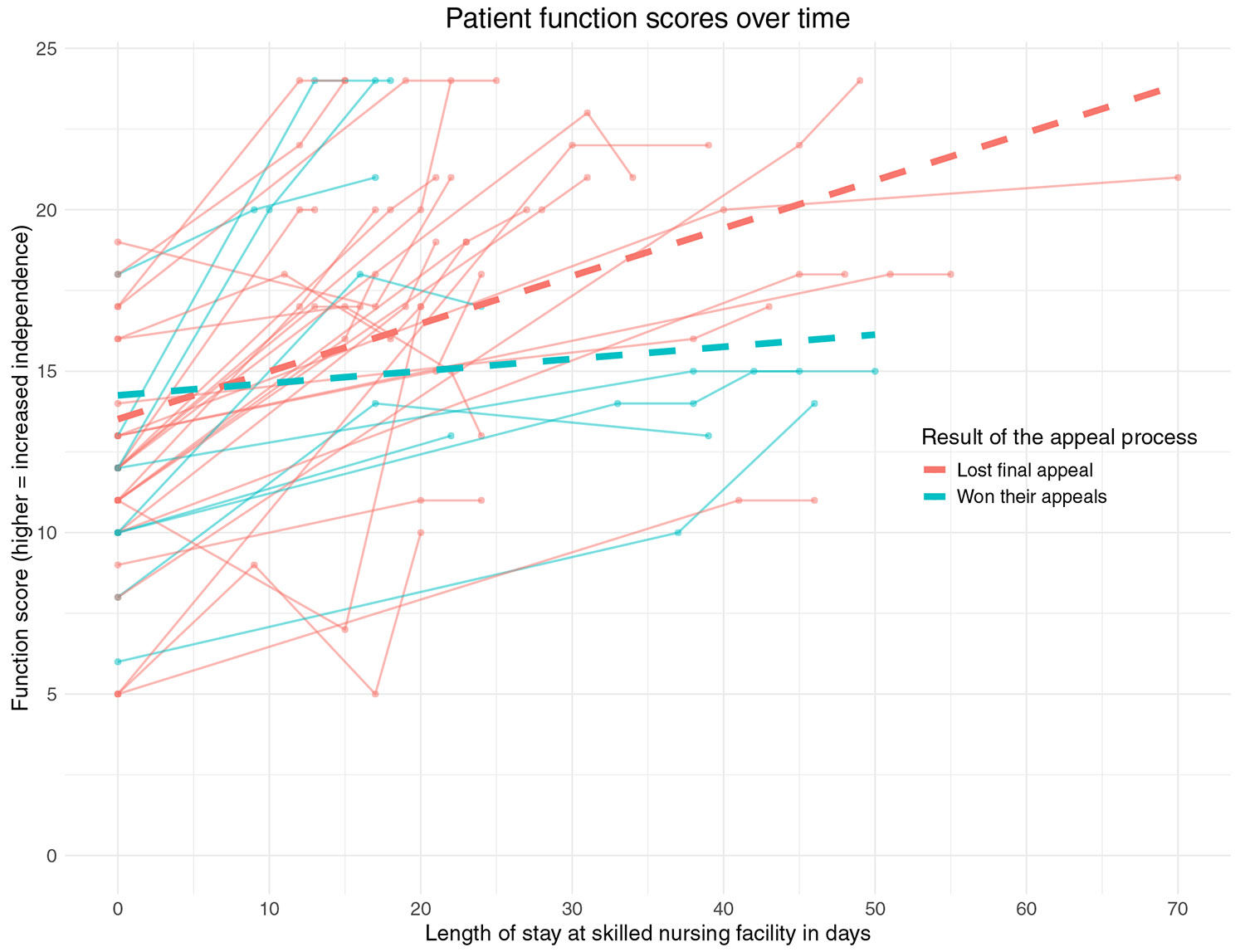
Function scores over time stratified by patients who lost their final appeal and those who won their appeals. The function score ranges from 0 to 24, with higher scores indicating increased independence. Each solid line represents the functional trajectory over time of an individual patient. The thick dotted lines represent the mean function scores among those who lost any appeal and those who won their appeals. The function score is based on the PDPM function score, which evaluates a patient’s level of functioning on the tasks of eating, oral hygiene, toileting hygiene, sit to lying, lying to sitting on side of bed, sit to stand, chair/bed-to-chair transfer, toilet transfer, walking 50 feet with 2 turns, and walking 150 feet. See the Supplementary Methods for additional details. A total of 36 of the 47 patients had functional assessment data available (27 patients who lost their final appeal and 9 patients who won their appeals).

Figure 2 shows function scores over time for the 36 individuals with available functional data, stratified by whether the patient lost their final appeal or won their appeals. Both groups had similar starting function scores with small improvements over time. Results were similar when analyzing by appeal (Supplementary Figure 2).

For most patients (n = 28, 59.6%), clinical notes documented a patient and/or family/caregiver concern about discharge readiness rather than clinician concern, with similar proportions among those who won their appeals and lost their final appeal.

## Discussion

In this retrospective cohort study of post-acute care patients at a single SNF in northern California, 10% of patients appealed their discharge dates, with more than half reporting concerns about discharge readiness. Most patients who appealed eventually lost (77%, n = 36/47). These findings, alongside preliminary data on outcomes such as functional status, rehospitalizations, and mortality, can help inform the design of larger-scale studies across multiple SNFs.

Our descriptive results have several implications. First, the fact that most appellants lost their final appeal and commonly reported ongoing concerns about discharge readiness highlights the significant distress experienced by appellants. This may reflect a broad disconnect between patient/caregiver expectations regarding discharge readiness and eligibility criteria for institutional skilled post-acute care set out in the Medicare Benefit Policy Manual.^13^ This disconnect transcended insurance type: we anticipated that nearly all appeals would originate from MA enrollees due to stricter scrutiny of SNF length of stay in MA programs^14-16^; however, many appeals originated from traditional Medicare patients (43%, n = 20 of 47), whose discharge dates are typically set by the SNF team. Many appellants likely have chronic care needs that may no longer require post-acute rehabilitation, but rather long-term services and support (LTSS), such as housekeeping, meal preparation, and personal care assistance.^4,17,18^ Unfortunately, LTSS are not covered under the post-acute care benefit and remain underfunded in the United States^19-23^; future research could examine whether the incidence of appeals is related to local LTSS availability.

Second, our findings highlight the importance of improving transitional care for the SNF-to-home transition. We found that patients who won their appeals gained an additional 4 SNF days without much additional functional improvement and still mostly went home. Appeals may be borne from a gap in patient/caregiver knowledge about how to manage medications, wound care, and maintenance therapy after discharge.^4^ Transitional care programs that focus on creating care plans before SNF discharge could help.^5,24,25^

Third, we noted that clinical documentation about appeals was sparse. If SNF staff believe patients have ongoing skilled needs, thorough clinical documentation about those needs—including the diagnosis and specific ongoing skilled nursing/therapy needs—is essential to effectively advocate for patients.

Our study has important limitations and ultimately lays the groundwork for future research. First, data were collected from a single northern California nonprofit SNF, which limits generalizability. Second, small sample sizes prevented us from drawing conclusions about differences in outcomes among patients who won or lost their appeals. Nevertheless, we found plausible differences in clinical outcomes, such as function, rehospitalization, and mortality, which warrant further investigation across multiple SNFs. Third, we relied on EHR documentation to ascertain the appeal reason. Future research would benefit from incorporating the interdisciplinary SNF team’s assessment of the merit of appeals based on their clinical knowledge. Fourth, we did not directly compare those who appealed with those who did not, which limits the assessment of differences between these 2 populations, although the rehospitalization and mortality rates in our cohort of appellants were comparable to national averages.^1,11,26^ Fifth, appeals may not capture all disputed discharges, as SNF clinicians and insurance companies may engage in peer-to-peer discussions to extend SNF stays without an appeal being filed; this requires documentation. Finally, although the EHR includes information from most hospitals in the surrounding region, we may have missed rehospitalizations to hospitals not linked to our academic hospital’s EHR. Future studies can link to information available in Medicare claims.

## Conclusions and Implications

Discharge appeals in this single northern California SNF were filed for 10% of patients and were frequently due to patient and/or family/caregiver concerns about discharge readiness. These findings lay the groundwork for future research across multiple SNFs to document appeal volume and success rates, identify when appeals are supported by clinician concerns as opposed to patient and/or family/caregiver distress alone, and examine disparities in outcomes among those who win or lose their appeals.

## Supplementary Material

1

Supplementary data related to this article can be found online at https://doi.org/10.1016/j.jamda.2025.106108.

## Figures and Tables

**Table 1:** Descriptive Characteristics and Outcomes of Patients Who Filed a Discharge Appeal During Their Short-Stay SNF Admission

	Overall (n = 47)	Lost Their Final Appeal (n = 36)	Won Their Appeals (n = 11)
Age, y, mean (SD)	79.3 (10.6)	77.4 (10.4)	85.5 (9.19)
Sex			
Female	25 (53.2)	17 (47.2)	8 (72.7)
Male	22 (46.8)	19 (52.8)	3 (27.3)
Race			
White	28 (59.6)	22 (61.1)	6 (54.5)
Asian	9 (19.1)	6 (16.7)	3 (27.3)
Black	7 (14.9)	7 (19.4)	0 (0)
Hispanic	1 (2.1)	0 (0)	1 (9.1)
Other*	2 (4.2)	1 (2.8)	1 (9.1)
Married	21 (44.7)	17 (47.2)	4 (36.4)
Medicaid enrolled	9 (19.1)	7 (19.4)	2 (18.2)
Insurance coverage			
Medicare Advantage	27 (57.4)	19 (52.8)	8 (72.7)
Traditional Medicare	20 (42.6)	17 (47.2)	3 (27.3)
Primary medical diagnosis^†^			
Orthopedic (eg, joint replacement, spinal surgery, fracture)	20 (42.6)	15 (41.7)	5 (45.5)
Acute neurologic	7 (14.9)	3 (8.3)	4 (36.4)
Cardiovascular and coagulation	7 (14.9)	6 (16.7)	1 (9.1)
Medical management	6 (12.8)	5 (13.9)	1 (9.1)
Acute infections	4 (8.5)	4 (11.1)	0 (0)
Pulmonary	2 (4.3)	2 (5.6)	0 (0)
Cancer	1 (2.1)	1 (2.8)	0 (0)
Hospital length of stay, median (IQR)	7.00 (4.00–11.5)	7.00 (4.75–12.0)	5.00 (4.00–10.0)
Comorbidities from Charlson index, mean (SD)	2.87 (1.65)	3.06 (1.71)	2.27 (1.35)
Function score on admission (range 0-24), mean (SD)^‡^	11.72 (3.61)	11.96 (3.72)	11 (3.39)
Cognitive impairment^§^	7 (14.9)	4 (11.1)	3 (27.3)
Urinary incontinence or catheter use	30 (63.8)	22 (61.1)	8 (72.7)
Bowel incontinence or ostomy	26 (55.3)	19 (52.8)	7 (63.6)
Number of appeals per person, mean (SD)	1.23 (0.60)	1.25 (0.60)	1.18 (0.60)
Outcomes			
Time from SNF admission to first appeal in days, median (IQR)	19.0 (15.0–30.5)	19.5 (15.0–30.25)	17.0 (14.5–31.0)
Time from first appeal to SNF discharge in days, median (IQR)	5.00 (3.00–9.00)	4.00 (3.00–8.00)	8.00 (7.00–13.0)
Total length of stay at SNF, median (IQR)	25.0 (20.0–39.0)	25.0 (20.75–37.25)	31.0 (19.0–45.5)
Discharge location			
Home	30 (63.8)	24 (66.7)	6 (54.5)
Long-term care transition (new)	8 (17.0)	6 (16.7)	2 (18.2)
Assisted living facility (new)	3 (6.4)	3 (8.3)	0 (0)
Home hospice	2 (4.3)	0 (0)	2 (18.2)
Hospitalization	2 (4.3)	2 (5.6)	0 (0)
Acute psychiatric unit	1 (2.1)	1 (2.8)	0 (0)
Death	1 (2.1)	0 (0)	1 (9.1)
30-day rehospitalization^‖^	10 (21.3)	10 (27.8)	0 (0)
30-day mortality^‖^	2 (4.3)	0 (0)	2 (18.2)**
3-month mortality^‖^	6 (12.8)	3 (8.3)	3 (27.3)
6-month mortality^‖^	8 (17.0)	5 (13.9)	3 (27.3)
Reason for appeal			
Patient and/or family/caregiver concern	28 (59.6)	22 (61.1)	6 (54.5)
SNF clinician concern	4 (8.5)	3 (8.3)	1 (9.1)
No reason documented	15 (31.9)	11 (30.6)	4 (36.4)

NR, not reported.

Values are n (%) unless otherwise noted.

* Other includes 1 patient who identified as Iranian and 1 patient who identified as Pacific Islander.

† Primary diagnosis was based on the PDPM categories.

‡ The function score was based on the PDPM function score, which ranges from 0 to 24, with lower scores indicating increased functional impairment. See the Supplementary Methods for additional details. A total of 36 patients had functional assessment data on admission (27 patients who lost their final appeal and 9 patients who won their appeals).

§ Cognitive status was based on the MDS Cognitive Function Scale (CFS), which uses the Brief Interview for Mental Status (BIMS) screener (scored from 0–15) or Cognitive Performance Scale (CPS) (scored from 0–6).

‖ Thirty-day rehospitalization data were missing in 1 patient who lost their final appeal. Thirty-day and 3-month mortality were missing in 2 patients who lost their final appeal. Six-month mortality was missing in 3 patients who lost their final appeal. Missingness was based on the absence of clinical events in the linked hospital EHR during these time frames.

** This category includes 1 patient who died while at the SNF after winning an appeal and 1 patient who won an appeal, was subsequently discharged on home hospice, and died 8 days after being discharged from the SNF.
