# Supplementary material for: Can I Stay, or Must I Go Now? A Cohort Study of Discharge Appeals in a Post-Acute Skilled Nursing Facility
Source: J Am Med Dir Assoc. Author manuscript; Available in PMC 2026 Apr 22. (PMC13101059; doi:10.1016/j.jamda.2025.106108)
Supplement: 1 [file NIHMS2163406-supplement-1.docx]

**Supplement**

**Supplementary Methods**

**Supplementary Figure S1:** Flow diagram of appeals included in this study

**Supplementary Figure S2:** Patient function scores over time grouped at the level of the appeal

**Supplementary Table S1:** Codebook for reasons for discharge appeal based on clinical documentation in the electronic health record

**Supplementary Table S2:** Outcomes grouped at the level of the appeal stratified by whether the appeal was won or lost

**Supplementary Table S3:** Information for patients who had multiple appeals during a short-stay skilled nursing facility stay

**Supplementary Methods**

Calculation of Function Score

We report a patient’s function score based off the Patient-Driven Payment Model (PDPM) function score which ranges from 0-24 with lower scores indicating increased functional impairment. The function score is based on a patient’s level of function with the tasks of eating, oral hygiene, toileting hygiene, sit to lying, lying to sitting on side of bed, sit to stand, chair/bed-to-chair transfer, toilet transfer, walking 50 feet with two turns, and walking 150 feet. Performance is graded as independent, setup/clean-up assistance, supervision/touching assistance, partial/moderate assistance, substantial/maximal assistance, and dependent/refused/not attempted.

The total score is taken as the sum of the eating function score, oral hygiene function score, toileting hygiene function score, average bed mobility function score (the average of sit to lying sore and lying to sitting on side of bed score), average transfer function score (the average of sit to stand, chair/bed-to-chair score, and toilet transfer score), and average walking function score (the average of walk 50 feet with two turns and walk 150 feet score).

At the skilled nursing facility (SNF) for this study, information on function scores is reported in physical and occupational therapy notes. Upon SNF admission, therapists conduct an admission assessment in which scores for all items are reported to calculate the function score. During the SNF stay, progress notes usually update the function scores each week. When a patient is discharged, a discharge summary note is written, which contains the patient’s functional status at the time of assessment closest to their discharge from the SNF. For this study, we extracted function scores from the admission assessment (performed within 1-2 days in all cases), from the progress note at the time of discharge appeal (performed within 0-3 days in all cases), and from the discharge assessment (typically performed within 1-2 days of either home discharge, custodial transition, or transfer to the hospital).

**Supplementary Figure S1:** Flow diagram of appeals included in this study

**
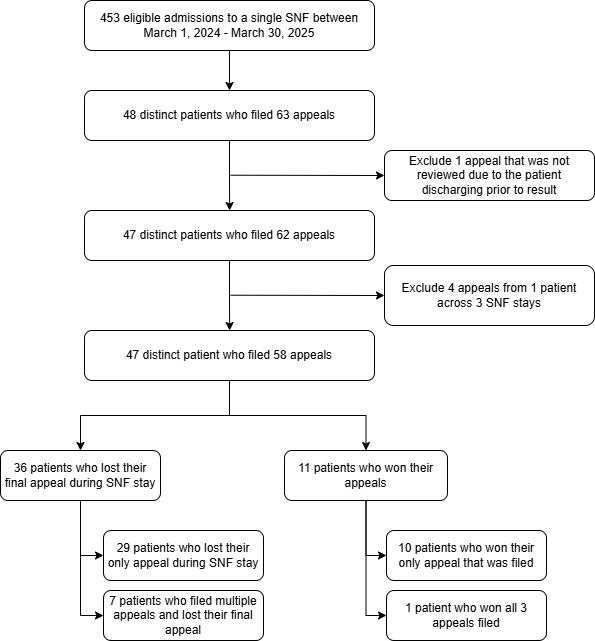
**

Abbreviations: SNF, skilled nursing facility

**Supplementary Figure S2:** Patient function scores over time grouped at the level of the appeal^a^

**
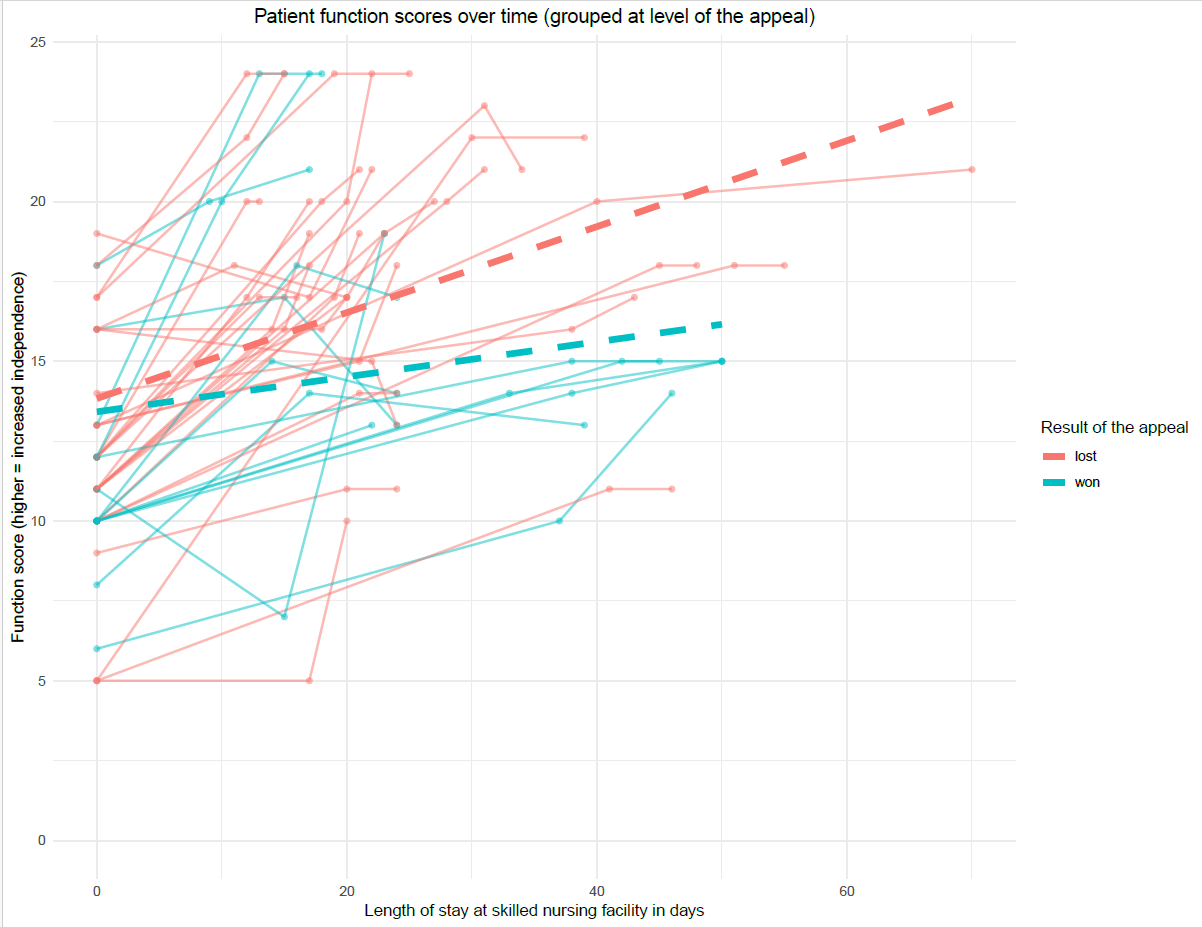
**

Note: In contrast to Figure 2, function scores in this Figure are for each appeal rather than for individual patients. In other words, patients with multiple appeals have multiple lines in this plot, whereas in Figure 2, each line represents one person. Scores were assessed at admission, the time of the appeal, and the time of discharge.

a The function score ranges from 0-24 with higher scores indicating increased independence. Each solid line represents the functional trajectory of an individual patient. The thick dotted lines represent the mean functional scores among those who lost any appeal and those who won their appeals. The function score is based on the Patient-Driven Payment Model (PDPM) function score which evaluates an patient’s level of functioning on the tasks of eating, oral hygiene, toileting hygiene, sit to lying, lying to sitting on side of bed, sit to stand, chair/bed-to-chair transfer, toilet transfer, walking 50 feet with two turns, and walking 150 feet. See Supplementary Methods for additional details. A total of 36 patients had functional assessment data upon admission (27 patients who lost their final appeal and 9 patients who won their appeals).

**Supplementary Table S1:** Codebook for reasons for discharge appeal based on clinical documentation in the electronic health record

| **Reason for appeal** | **Code** | **Example** |
| --- | --- | --- |
| No reason documented | No documentation found in the electronic health record | No note in the chart |
|  | Documentation only indicated that the appeals process was being followed | Documentation that the patient was notified of their right to appeal and that they filed an appeal |
| Patient and/or family/caregiver concern | In addition to details about the process being followed, clinical documentation explains that the patient and/or family/caregiver is concerned about their discharge readiness | Patient and or family/caregiver concerned about functional status (e.g., limited mobility), safety at home (e.g., due to stairs), or lack of support (e.g., caregiver not able to provide support during the day due to work) |
| Clinician concern | Clinical documentation indicates SNF staff are concerned about discharge | Patient is still max assist |
|  | Clinical documentation indicates a change in condition | New-onset encephalopathy or hematuria |

**Supplementary Table S2:** Outcomes grouped at the level of the appeal stratified by whether the appeal was won or lost

|  | **Lost appeal (N=37)** | **Won appeal (N=21)** | **Overall (N=58)** |
| --- | --- | --- | --- |
| **Time from SNF admission to appeal in days, median (IQR)** | 21.0 (17.0 – 33.0) | 18.0 (15.0 – 29.0) | 20.5 (15.3 – 32.5) |
| **Time from appeal to SNF discharge in days, median (IQR)** | 3.00 (3.00 – 5.00) | 9.00 (8.00 – 12.0) | 5.00 (3.00 – 9.00) |
| **Total length of stay at SNF, median (IQR)** | 25.0 (20.0 – 37.0) | 35.0 (23.0 – 45.0) | 27.5 (20.3 – 38.75) |

Abbreviations: IQR, interquartile range; SNF, skilled nursing facility

**Supplementary Table S3**: Information for patients who had multiple appeals during a short-stay skilled nursing facility stay

|  | **Lost final appeal**  **(N=7)** | **Won their appeals**  **(N=1)** | **Overall**  **(N=8)** |
| --- | --- | --- | --- |
| Number of appeals, median (IQR) | 2 (2 – 2) | 3 (3 – 3) | 2 (2 – 2.25) |
| Time to first appeal in days, median (IQR) | 15.0 (13.0 – 16.5) | 33.0 (33.0 – 33.0) | 15.0 (14.0 – 20.75) |
| Time between appeals in days, median (IQR) | 7.00 (6.00 – 7.00) | 4.50 (4.25 – 4.75) | 6.00 (5.00 – 7.00) |
| Total SNF length of stay in days, median (IQR) | 24.0 (21.5 – 32.5) | 50.0 (50.0 – 50.0) | 27.0 (22.25 – 35.75) |

Abbreviations: IQR, interquartile range; SNF, skilled nursing facility

Note: Of the 7 patients who filed multiple appeals and lost their final appeal, 5 patients won 1 appeal and lost the second appeal, 1 patient lost both their appeals, and 1 patient won 3 appeals and lost the 4^th^ appeal. The patient who won all their appeals filed 3 appeals.
